# Deciphering genotype-by-environment interaction of grass pea genotypes under rain-fed conditions and emphasizing the role of monthly rainfall

**DOI:** 10.1186/s12870-024-05256-5

**Published:** 2024-06-15

**Authors:** Hamid Hatami Maleki, Behrouz Vaezi, Askar Jozeyan, Amir Mirzaei, Reza Darvishzadeh, Shahryar Dashti, Hossein Abdi, Hossein Zeinalzadeh-Tabrizi

**Affiliations:** 1 Department of Plant Production and Genetics, Faculty of Agriculture, University of Maragheh, Maragheh, Iran; 2 Kohgiluyeh and Boyerahmad Agricultural and Natural Resources Research and Education Center, Agricultural Research, Education and Extension Organization (AREEO), Yasuj, Iran; 3 Ilam Agricultural and Natural Resources Research Center, Agricultural Research, Education and Extension Organization (AREEO), Ilam, Iran; 4 Department of Plant Production and Genetics, Faculty of Agriculture, Urmia University, Urmia, Iran; 5 Department of Horticulture and Agronomy, Faculty of Agriculture, Kyrgyz-Turkish Manas University, Bishkek, Kyrgyzstan

**Keywords:** Seed and forage yield, Multi-trait stability index, Partial least square regression, WAASB stability analysis

## Abstract

Rainfed regions have inconsistent spatial and temporal rainfall. So, these regions could face water deficiency during critical stages of crop growth. In this regard, multi-environment trials could play a key role in introducing stable genotypes with good performance across several rainfed regions. Grass pea, as a potential forage crop, is a resilient plant that could grow in unsuitable circumstances. In this study, agro-morphological attributes of 16 grass pea genotypes were examined in four semi-warm rain-fed regions during the years 2018–2021. The MLM analysis of variance showed a significant genotype-by-environment interaction (GEI) for dry yield, seed yield, days to maturity, days to flowering, and plant height of grass pea. The PLS (partial least squares) regression revealed that rainfall in the grass pea establishment stage (October and November) is meaningful. For grass pea cultivation, monthly rainfall during plant growth is important, especially in May, with an aim for seed yield. Regarding dry yield, G5, G10, G11, G12, G13, and G15 were selected as good performers and stable genotypes using DY × WAASB biplots, while SY × WAASB biplot manifested G2, G3, G12, and G13 as superior genotypes with stable seed yield. Considering equal weights for yield as well as the WAASB stability index (50/50), G13 was selected as the best one. Among test environments, E2 and E11 played a prominent role in distinguishing the above genotypes from other ones. In this study, MTSI (multi-trait stability index) analysis was applied to select a stable genotype, considering all measured agro-morphological traits simultaneously. Henceforth, the G5 and G15 grass pea genotypes were discerningly chosen due to their commendable performance in the WAASBY plot. In this context, G13 did not emerge as the winner based on MTSI; however, it exhibited an MTSI value in close proximity to the outer boundary of the circle. Consequently, upon comprehensive consideration of all traits, it is deduced that G5, G13, and G15 can be appraised as promising superior genotypes with stability across diverse environmental conditions.

**Supplementary Information:**

The online version contains supplementary material available at 10.1186/s12870-024-05256-5.

## Introduction

*Lathyrus sativus* L. commonly recognized as grass pea, cicerchia, blue sweet pea, chickling pea, chickling vetch, Indian pea, white pea, and white vetch, belongs to the legume family and boasts a rich agricultural heritage. Its cultivation has been deeply rooted in various regions of Iran, serving as a staple for both human and animal consumption, utilized both as forage and grain [1]. Grass pea, characterized by its brief growth cycles adaptable to both cold and hot seasons, stands out as a hardy plant capable of thriving in arid conditions, thereby demonstrating resilience in unfavorable environments. Its advantageous features, including elevated yield, a notable protein content, nitrogen-fixing abilities, and tolerance to drought, salinity, and waterlogging, underscore its significance in facilitating crop rotation, enhancing soil quality, and mitigating challenges associated with weeds, pests, and diseases [2]. These attributes position it as an exceptional crop, contributing significantly to ensuring nutritional security, particularly in anticipation of impending climate challenges [3]. In addition, considering the vast distribution of dryland regions around the globe [4], producing promising genotypes with high forage and seed yields under such conditions would assist the livestock and poultry industries. In arid conditions, the quantity and distribution of rainfall stand as primary limiting factors [5]. The introduction of grass pea cultivars tailored to these constraints holds substantial potential for enhancing both biological and grain yield. Beyond the compatibility of a specific grass pea cultivar, achieving yield stability across diverse environments is crucial. The significant impact of climatic and edaphic conditions on grass pea yield is well-established, with a notable genotype by environment interaction, as reported for its yield [6].

Yield stability refers to the consistent performance of a given genotype across various environments over multiple years. In the pursuit of introducing new varieties, the evaluation of genotype yield occurs through multi-environment trials (MET) conducted in diverse settings [7]. The presence of interaction between genotype and environment, especially in the case of complex traits such as yield, slows down the process of selecting genotypes and introducing new varieties. Therefore, the correct interpretation of this interaction is of fundamental importance in the process of evaluating and identifying superior genotypes. A suite of univariate as well as multivariate statistical methods has been introduced for the interpretation of genotype × environment interaction. As a multivariate method, the additive main effects and multiplicative interaction (AMMI) method, as described by Gauch [8], has been extensively utilized for the analysis of yield stability in various crops, notably in the case of grass pea [9]. In conjunction with the AMMI method, the best linear unbiased prediction (BLUP) method has been employed to assess both the adaptability and stability of targeted genotypes in multi-trial scenarios. While both AMMI and BLUP aim to extract genotype-by-environment interaction from random error, they differ in their underlying nature. Gauch [10] demonstrated that AMMI analysis captures the majority of the G × E pattern in the first interaction principal component axis (IPCA), with most random error being accounted for in the subsequent IPCAs. Concurrently, the BLUP method evaluates the genetic merit of the studied genotypes by estimating their mean yield in mixed models with high efficiency [11]. Building upon the existing literature, Olivoto et al. [12] introduced a novel approach termed weighted average absolute scores of BLUPs (WAASB), which combines elements from both the AMMI and BLUP methods. In essence, singular value decomposition is applied to a BLUP matrix, facilitating the analysis of genotype × environment interaction within a linear mixed model (LMM) framework. Subsequently, the WAASBY biplot, depicting the interaction of WAASB with the trait mean (Y), offers a comprehensive interpretation of both stability and trait productivity. Recognizing the importance of considering traits related to yield alongside yield measurements in the variety realization process, Olivoto et al. [13] introduced the MTSI (Multi-Trait Stability Index). Calculated based on the distance from the ideal genotype estimated through factor analysis, the MTSI index has demonstrated efficacy in various crops, including guar [14], maize [15, 16], lentil [17], sugar beet [18], and oilseed rape [19]. While there is no existing report on the application of the MTSI index in grass pea stability analysis, its successful application in other crops underscores its potential utility in this context.

The interaction between genotype and environment has been extensively explored by researchers focusing on grass pea, with a predominant emphasis on economic aspects, particularly yield [20, 21]. Previous stability analyses in grass pea have predominantly employed univariate approaches [22] and relevant multivariate analyses such as AMMI [9] and GGE biplot analysis [21]. However, there is limited knowledge regarding the stability analysis of grass pea using WAASB, and little attention has been given to the simultaneous measurement of a diverse array of agro-morphological traits. This study aims to fill this gap by evaluating the agro-morphological responses of 16 grass pea genotypes across four rainfed regions in Iran over three consecutive years. The objectives are to: i) identify the monthly rainfall influencing grass pea performance in rainfed conditions, and ii) assess the efficacy of WAASB and MTSI in identifying high-productivity and stable grass pea genotypes well-adapted to these regions.

## Material and methods

### Plant materials

The plant materials used comprised seven grass pea inbred lines originating from Greece, Hungary, Nepal, Morocco, and Bangladesh, along with seven inbred lines of unknown origin. Additionally, one grass pea line from ICARDA (International Center for Agricultural Research in the Dry Areas) and one inbred line (serving as a control) from Iran were included in the study (Table 1). This germplasm was sourced from the gene bank section of DARI (Dryland Agricultural Research Institute) in Iran.

**Table 1:** Code and origin of grass pea genotypes used in the multi-environment experiments

Number	Code	Origin	Number	Code	Origin
G1	–	–	G9	IFLA No.127	Greece (GRC)
G2	IFLA No.1961	Nepal (NPL)	G10	PN = 223	–
G3	IFLA No.2990	Bangladesh (BGD)	G11	PN = 225	–
G4	IFLA No.1847	Bangladesh (BGD)	G12	PN = 226	–
G5	IFLA No.2968	Bangladesh (BGD)	G13	PN = 219	–
G6	IFLA No.1707	Morocco (MAR)	G14	PN = 222	–
G7	IFLA No.Bio520	ICARDA	G15	PN = 224	–
G8	IFLA No.276	Hungary (HUN)	G16	Naghadeh	Iran

### Environments and experimental design

**Fig. 1:**
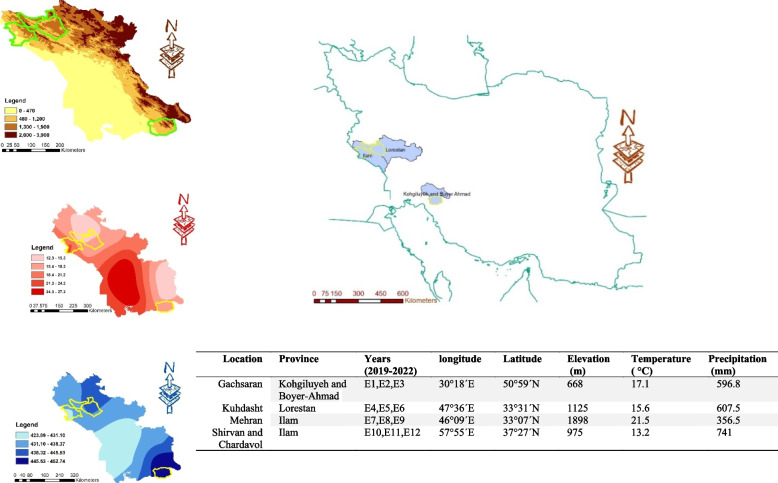
Geographical coordinates, agro-climatic characteristic of test locations. The map constructed by using elevation, temperature, and precipitation information of presented environments in ArcGIS 10.8 software

The field study was conducted at four locations, Gachsaran, Mehran, Kuhdasht, Shirvan and Chardavol, over three consecutive years (2018–2019, 2019–2020, and 2020–2021). These locations, situated in three geographically diverse provinces of Iran, were categorized as semi-warm regions based on climatic attributes (Fig. 1). The physical and chemical properties of studied region`s soil was presented in Table S1. For each environment (year × location), the experimental design employed a randomized complete block design (RCBD) with three replications. Each plot consisted of four rows, each 4.5 m in length, with a spacing of 25 cm between rows. The seeding rate was maintained at 150 seeds per m^^^2 across all environments. Field practices, including weed control, were executed manually during crop growth and development.

### Measured traits

During the growing season, various traits such as days to flowering (DF), days to maturity (DM), and plant height (PH) were recorded. At the time of harvest, traits such as number of pods per plant (PP), number of seeds per pod (SP), wet fodder yield (WY), dry fodder yield (DY), and grain yield (GY) were measured. GY measurements were obtained by harvesting the central four rows for each line in all experiments. WT and DY at 50% flowering stage and grain yields at physiological maturity (ton ha^−1^) were determined by converting the yields obtained from the plots to hectares.

### Data analysis

The data was tested for normality by the Anderson–Darling test and checked for outliers, then Levene's test was used for the homogeneity of variance test to confirm the homogeneity of individual error mean squares. To evaluate the genotypes' stability across the environments, a linear mixed model was used [13]. Accordingly, the significance of each effect for the studied traits was tested by the likelihood ratio test (LRT) with a two-tailed chi-square test with one degree of freedom. So, for each environment, the traits were initially fitted into a linear mixed-effect model by considering environment and environment-by-genotype interaction as random effects and genotype as a fixed effect [12]. The following standard linear mixed model [11] was computed with the function “gamem_met” from the metan R-package [13].

$$\mathrm y\;=\;\mathrm{Xb}\;+\;\mathrm{Zu}\;+\;\in$$

where y is a vector of response variable, b is a vector of fixed effects, u is a vector of random effects, X is a design matrix of 0s and 1s relating y to b, Z is a design matrix of 0s and 1s relating y to u, and ϵ is a vector of random errors.

After analysis of variance, it is assumed that genotype and GEI are random effects [12] to predict genetic parameters using the argument “genpar” in the function gamem_met. Then, stability analysis was exerted by the calculation of WAASBi using the function “waasb” in the metan package. In this process, WASSB was estimated based on a single value decomposition of the G × E interaction effects from the matrix of the BLUP as follows:

$${WAASB}_{i}=\frac{\sum_{k=1}^{p}|{IPCA}_{ik}\times {EP}_{k}|}{\sum_{k=1}^{p}{EP}_{k}}$$

where WAASBi is the weighted average of absolute scores of the i*th* genotype or environment, IPCA_ik_ is the absolute score of the i*th* genotype or environment in the k*th* IPC, and EP_k_ is the magnitude of the variance explained by the k*th* IPC.

As shown, WAASBYi is the superiority index with different weights between yield and stability for the g*th* genotype, Ɵ_Y_ and Ɵ_S_ are the weights for yield and stability, respectively; rG_g_ and rW_g_ are the rescaled values of the g*th* genotype for yield and WAASB, respectively.

$$WAASBY_i\;=\;\frac{\left(rG_g\;\times\;\theta_Y\right)\;+\;\left(rW_g\;\times\;\theta_S\right)}{\theta_Y\;+\;\theta_S}$$

In the present study, MTSI was applied to calculate the mean performance and simultaneous stability of traits having significant G × E interaction comprising DM, PH, DF, DY, and SY, considering that higher values for studied traits except DM are suitable. In this regard, the vector of trait importance as c (l, h, h, h, h) was defined and incorporated into the WAASB analysis before the MTSI approach [14]. Then, MTSI analysis was done by the function MTSI in package metan as follows:

$$MTSI_i=\lbrack\sum\limits_{j=1}^f{(^\gamma ij\,-\,^\gamma j)}^2\rbrack^{0.5}$$

Where MTSIi is the multi-trait stability index of the genotype i, 𝛾ij is the score of the genotype i in the factor j, and 𝛾j is the score of the ideal genotype in the factor j. Scores were calculated based on factor analysis for genotypes and traits.

Incorporating rainfall as a covariate for explaining the GEI was done by using partial least squares (PLS) regression analysis in GEA-R software [23]. Hence, monthly rainfall from October to May is regarded as an environmental covariable. The PLS model consists of an independent matrix X (rainfall data), a dependent matrix Y (yield), and the latent variables t as follows:

$$x={t}_{1}{p{\prime}}_{1}+{t}_{2}{p{\prime}}_{2}+\dots +E=T{P}{\prime}+E$$

$$y={t}_{1}{q{\prime}}_{1}+{t}_{2}{q{\prime}}_{2}+\dots +E=T{Q}{\prime}+E$$

where matrix T contains X-scores, matrix P contains the X-loadings, matrix Q contains the Y-loadings, and F and E are the residual matrices. Finally, PLS results were presented in the form of a biplot.

## Results

**Fig. 2:**
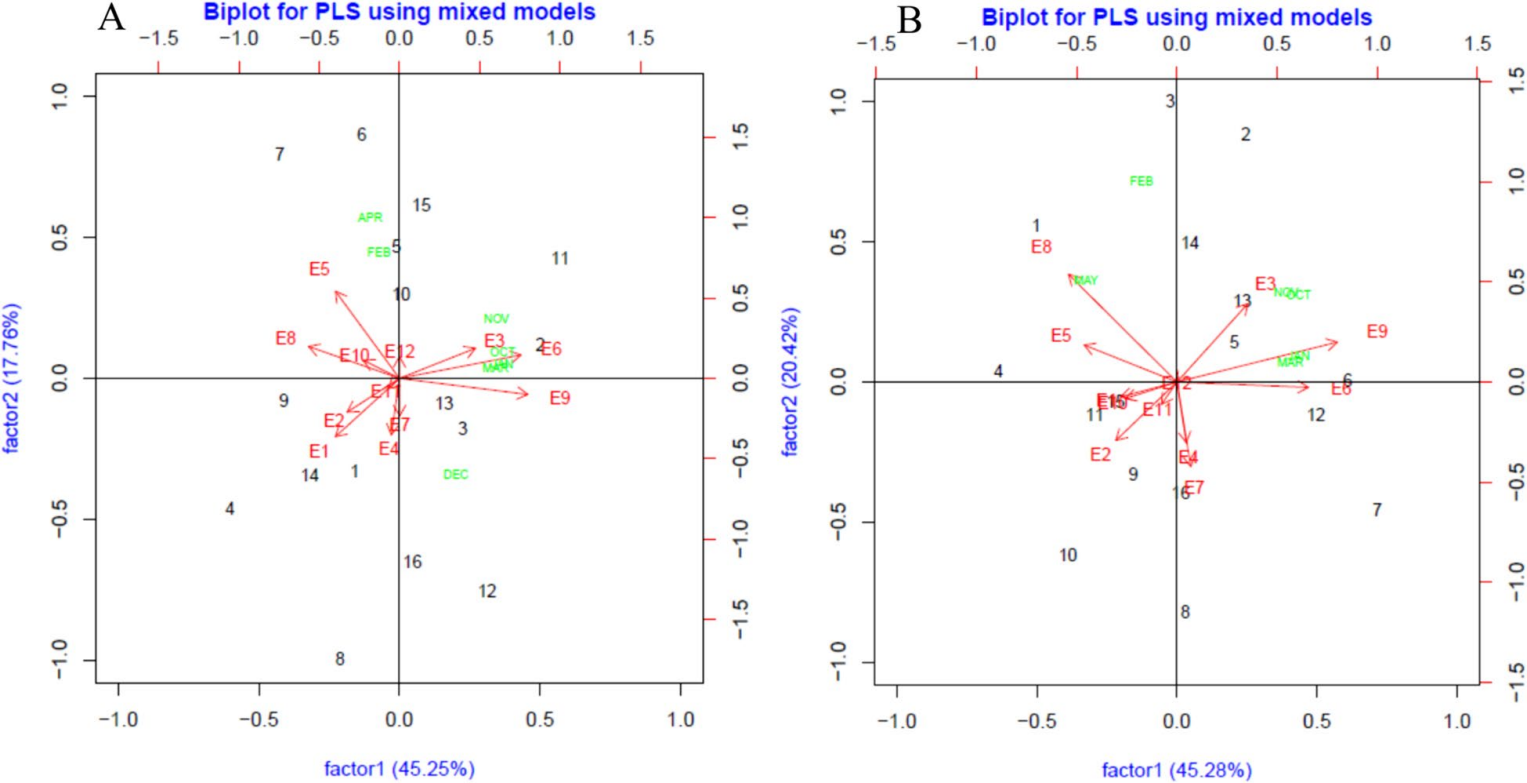
Biplot based on PLSR method with months’ rainfall as covariates for DY (**A**) and SY (**B**) of 16 grass pea genotypes in 12 environments. In each plot, the environments depicted by "E" and each months depicted with green color

It was inferred from ordinary analysis of variance (Table S2) and LRTe (Table 2) that the environment effect is highly significant for all of the studied traits, and so there is a difference among the tested environments. The GEI was significant for the majority of traits except PP, SP, and WY, according to LRTge. The phenotypic variance varied between 0.304 (SY) and 57.800 (PH). Both the genotypic and residual coefficients of variation showed variation for the studied traits. In this study, the coefficient of variation of GEI (Table 2) interaction as an indicator of trait reaction in response to environment was low for traits with non-significant GEI. Herein, rainfall during the grass pea growth period is considered a covariate through PLS regression to identify effective monthly rainfall that impacts DY, SY, and their GEI (Fig. 2A and B). For DY (Fig. 2A), the first and second factors in the PLS biplot explained 45.25% and 17.76% of the GEI variance, while for SY (Fig. 2B), factor 1 and factor 2 interpreted 45.28% and 20.425% of it. Regarding the eight months from planting (October) to harvest time (May), rainfall in all months except May for DY and December and April for SY had remarkable effects on GEI. In detail, rainfall in April and February is important for the dry yield of genotypes G5 and G15; rainfall in October, November, and March was meaningful for G2; and also, December rainfall has a greater contribution to G3 (Fig. 2A). Among the test environments, the highest values of monthly rainfall for October, November, January, and March were seen for E3, E6, and E9, while E5 had the highest value of monthly rainfall for February and April (Fig. 2A). Considering seed yield (Fig. 2B), most genotypes had no significant relationship with monthly rainfall, except for G5, G6, G12, and G13, which were affected by October, November, January, and March monthly rainfall. The E3, E6, and E9 had the highest values of rainfall in October, November, January, and March, while the E8 had the maximum value of rainfall in May (Fig. 2B).

**Table 2:** Likelihood ratio test (LRT) values, and genetic parameters for agro-morphological traits of 16 grass pea genotypes across 12 environment

Trait	LRT_e_	LRT_ge_	${\sigma }_{p}^{2}$	${CV}_{g}$	${CV}_{r}$	GEIr^2^
DF	891.000^**^	2.790^**^	9.520	0.213	2.160	0.293
DM	830.000^**^	3.550^**^	27.400	0.000	3.100	0.127
PH	402.000^**^	8.230^**^	57.800	1.630	11.700	0.142
PP	188.000^**^	2.900^ ns^	40.500	0.000	27.300	0.053
SP	1.530^**^	0.003^ ns^	1.230	2.970	31.800	0.001
WY	153.000^**^	0.074^ ns^	16.000	2.490	29.900	0.005
DY	4.050^**^	0.125^**^	0.995	6.170	22.300	0.126
SY	0.618^**^	0.068^**^	0.304	5.990	31.200	0.223

^**^significant at 1% (*p* < 0.01), ^*^significant at 5% (*p* < 0.05), *ns* nonsignificant, *LRTe* and *LRTge* Likelihood ratio tests for environment and genotype-by-environment interaction, respectively; phenotypic variance, *GEIr2* the coefficient of variation for GEI effects, *CVg* and *CVr* are genotypic and residual coefficient of variation respectively, *DF* days to flowering, *DM* days to maturity, *PH* plant height, *PP* number of pods per plant, *SP* number of seeds per pod, *WY* wet fodder yield, *DY* dry fodder yield, and *GY* grain yield

**Fig. 3:**
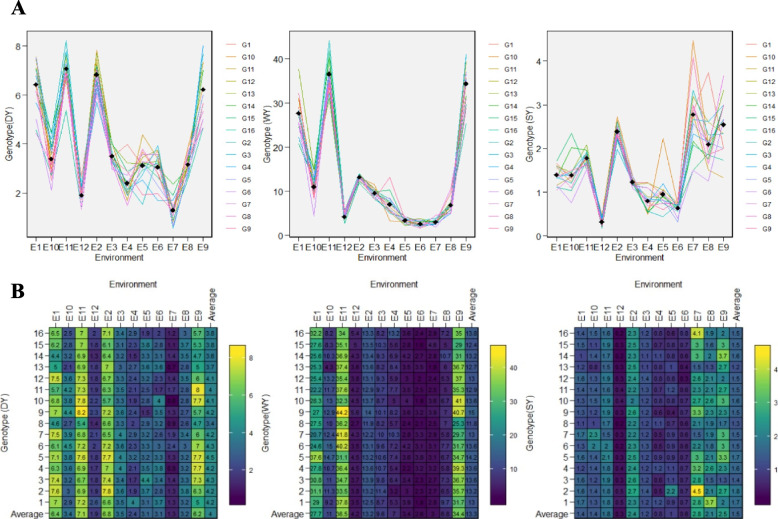
Genotype × environment plot of 16 grass pea genotypes in 12 test environments for DY (**A**), WY (**B**), and SY (**C**). Above plots showed fluctuation of yield across environments while below plot showed raw data recorded for each genotype across environments

The fluctuation of economic parts of grass pea including DY, WY, and SY, across test environments (Fig. 3A), was also implied by the existence of GEI for the mentioned traits. As shown in Fig. 3A, environments E3, E4, E5, and E6 jointly have no remarkable role in producing GEI for DY, WY, and SY. Regarding DY, WY, and SY traits, the average performance of each grass genotype in each of the test environments, the total average performance of each genotype in all environments, as well as the average performance of each environment, are presented in Fig. 3B. Overall genotype performance was varied among test environments, which verify GEI. The maximum values of DY and WY were detected for G9 in E11, while the maximum value of SY was seen in E7 for G2. Also, the highest mean performance for DY and WY belonged to E11, and considering SY, it was detected for E7. Among the studied genotypes, G5 with DY = 4.5, G9 with WY = 15, and G2 with SY = 1.8 had the maximum performance through test environments (Fig. 3A and B).

**Fig. 4:**
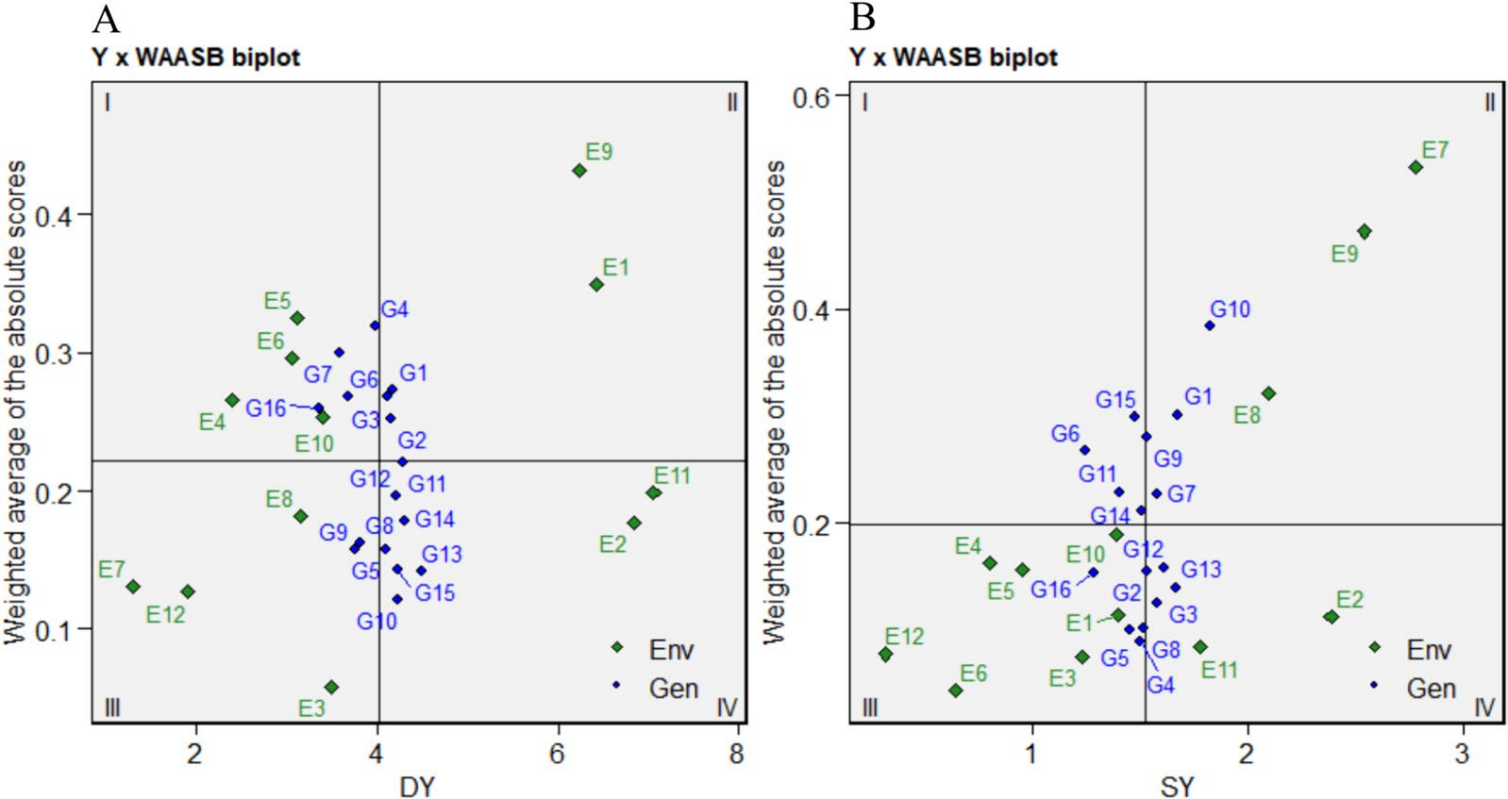
Yield × WAASB biplot for DY **A** and SY **B** as responsible variable across 12 environments. Each plot devided to four sections and in each plot, genotypes with good stability as well as yield performance higher than mean is located in section IV

The differing performance of studied grass pea germplasm across several environments makes it mandatory to identify high-yielding as well as stable genotypes to deliver for all of the studied rainfed locations. Hence, by utilizing DY and SY as responsible variables against WAASB values (Fig. 4A and B), the grass pea genotypes with stable performance could be distinguished. In the biplot of DY × WAASB (Fig. 4A), the first quarter serves as an indicator of both unstable and low-yield genotypes and environments. Therefore, genotypes G4, G6, G7, and G16 associated with environments E4, E5, E6, and E10 were identified. Similarly, the heatmap concerned with DY (Fig. 3A) also showed low mean values for the above-mentioned environments. In the second quarter, genotypes G1, G2, and G3 had a higher average performance than the overall average performance, but they had low WAASB values (Fig. 4A). This means that E1 and E9 (Figs. 3A and 4A) are good discriminators for genotypes like those located in quarter two. Here, some genotypes, such as G8 and G9, had poor DY but were stable (quarter three). As shown in Fig. 4A, genotypes G5, G10, G11, G12, G13, and G15 with low values of the WAASB stability index as well as high DY could be considered the best ones, and environments E2 and E11 played a key role in distinguishing these genotypes from other ones. About the SY trait (Fig. 4B), the distribution of the genotypes and test environments in the two-dimensional space of the SY × WAASB plot was varied compared with the DY × WAASB. In the SY × WAASB biplot, G2, G3, G12, and G13 (in quarter four) possessed low WAASB values and also performed better than the overall mean. The genotypes G4, G5, G8, and G16 were detected as stable but with low yield genotypes. Considering Fig. 4B, G1, G7, and G10 were unstable genotypes, with SY greater than the overall mean performance.

**Fig. 5:**
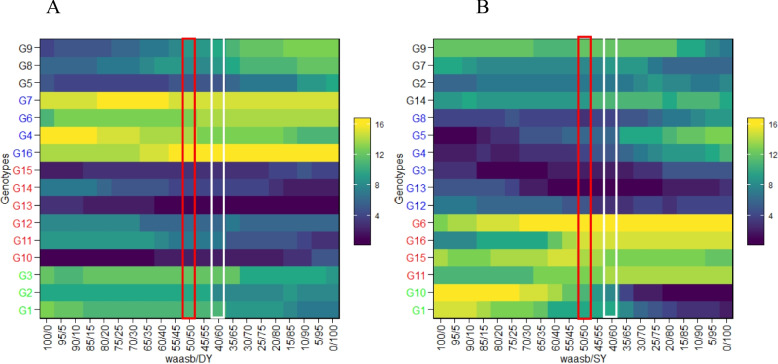
Heatmap showing the rank of 16 studied grass pea genotypes based on different weights for DY **A** and SY **B** versus WAASB stability index. The red rectangular (50/50) and white rectangular (60/40) shows that with change in weights of yield and stability the genotypes`s rank could be varied

**Fig. 6:**
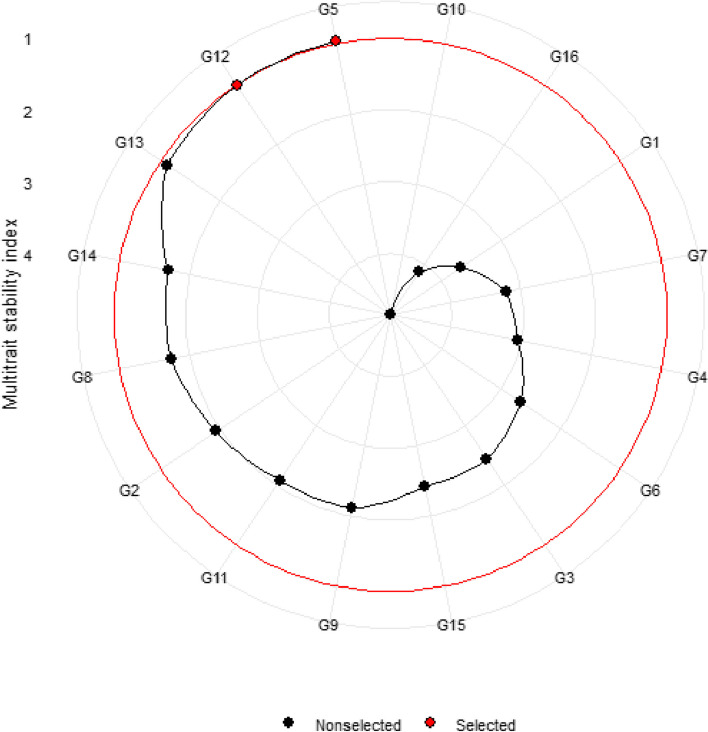
Genotypes ranking based on the multi-trait stability index. In this graph, the multi-trait stable genotypes signed with red point

To customize the magnitude of stability index and yield performance in identifying interest genotypes, plotting WAASB values against the responsible variable (WAASBY) was done regarding several weights for each WAASB and yield across test environments (Fig. 5A and B). Accordingly, a change in the ranking of genotypes considering the weight of DY and GY traits and the stability index (WAASB) was presented (Fig. 5A and B). In the first column on the left side (Fig. 5A and B), the ranking of genotypes based solely on the WAASB index (0/100) indicated that G5 and G10 were the most stable genotypes for DY and SY, respectively. In the last column on the right side, the rankings of genotypes were based solely on grain yield (100/0), making G13 and G10 the most superior genotypes regarding DY and SY, respectively. The red rectangle (Fig. 5A, B) is ranking the genotypes based on the equal weight for stability and the responsible variable (DY and GY), which is similar to the ranking of genotypes in Fig. 4A and B. In detail, G10, G13, and G15 were the best genotypes when DY with stability had equal weights (50/50), while G3, G12, and G13 were the superior genotypes based on GY in that selected condition. In grass pea, DY and GY are economic parts of the plant influenced by several agro-morphological traits, and hence, plant breeders frequently try to incorporate several traits into a new genotype to achieve a high yield. For achieving this, MTSI was computed based on simultaneous usage of DY and SY and other agro-morphological traits that had significant GEI, including DF, DM, and PH. Factor analysis after scaling the trait using BLUP for genotype mean performance resulted in two factors with eigenvalues greater than 1 that explained 76.7 percent of total variation (Table 3). This suggests that the two factors were successful in capturing a substantial amount of variability in the traits. Moreover, the communality values for the variables ranged from 0.688 for the DY trait to 0.813 for the DF trait, with a mean of 0.767. These values suggest that a significant portion of the variability of each variable was explained by these factors. Considering the loading coefficients in correspondence to each trait (Table 3), the studied agro-morphological attributes of the grass pea panel could be classified. Hence, in FA1 and FA2, traits DF and DY had positive loadings, while traits PH, DM, and SY possessed negative loadings. In Fig. 6, the experimental genotypes are ranked from the highest to the lowest value of the MTSI, so that the genotype with the highest value of the MTSI is in the center and the genotype with the lowest value of the MTSI is located in the outermost circle. The genotypes determined by the red dots were selected based on their MTSI values at 10% selection intensity. G12 was in the first rank, followed by G10, as the most ideal stable genotype. The average value of all traits except DM in selected genotypes has increased, which was aimed at the intended goals. In general, the selected genotypes caused a favorable selection differential in all traits (Table 4).

**Table 3:** Factorial loadings and communalities obtained from the factor analysis

Trait	FA1	FA2	Communality
DF	0.0182	0.901	0.813
DM	-0.878	-0.0723	0.776
PH	-0.801	-0.412	0.811
DY	0.22	0.8	0.688
SY	-0.864	-0.024	0.748

**Table 4:** Selection differential for the waasb index (up section) as well as mean of the traits (down section)

Trait	Factor	Xo	Xs	SD	Goal
DM	FA1	158	158	-0.0816	decrease
PH	FA1	59.5	59.7	0.268	increase
SY	FA1	1.53	1.54	0.00896	increase
DF	FA2	120	120	0.0538	decrease
DY	FA2	4.03	4.16	0.125	increase

## Discussion

The cultivation and advancement of forage legumes that are well-adapted to rainfed conditions present a promising perspective for rainfed crop rotation and forage supply. Opting to grow forage legumes in rainfed regions, instead of leaving the land fallow, holds the potential to enhance soil organic and nitrogen content. This, in turn, could contribute to an increase in the subsequent cereal yield cultivated in the rotation [24]. In this regard, grass pea as a forage legume with a short growth period as well as high compatibility with unsuitable circumstances has been reported [25]. In the present study, seed yield, dry yield, fresh yield, and agro-morphological traits of grass pea were evaluated for 3 years in 4 rainfed regions, which were calculated as semi-warm rainfed climates [20]. The tested environments differed from each other and significantly affected all of the grass pea traits. It is clearly inferable from the geographical attributes of selected regions that the elevation, temperature, and precipitation of the studied areas are varied. Accordingly, these regions were applied in most adaptability and stability studies handled by DARI as hot points for semi-warm rainfed studies. Studying GEI in grass pea revealed significant effects on all investigated agro-morphological traits, including seed yield and dry yield. The resulted GEI for the majority of traits manifests that genotypes ranking in different environments could be varied. Significant GEI for yield of grass pea was reported in some research works [9, 20–22, 26]. However, there are narrow studies about the evaluation of agro-morphological traits of grass pea in multi-trials.

Regardless of the aim of grass pea planting—dry yield or seed yield—the PLS regression analysis found that rainfall in October and November could influence grass pea establishment in rainfed environments. So it is concluded that while rainfall in rainfed conditions holds greater significance [27, 28], the distribution of rainfall during critical stages of crop growth in dryland conditions [27, 29] also proves to be crucial. In rainfed conditions, the precipitation in the last month of the growing period is important for the production of grass pea seeds, while with the aim of raising grass pea dry yield, having rainfall in the months leading up to the final month is more effective. Another application of PLS regression is in determining which month`s rainfall contributed the most to a given genotype yield and also which environment has the highest value of monthly rainfall [30]. In accordance with previous studies [27], PLS regression could effectively distinguish genotypes as well as environments in grass pea. For instance, E3, E6, and E9 have suitable values of precipitation in October, November, January, and March, which is prerequisite for achieving remarkable dry yield and seed yield in the studied grass pea germplasm under rainfed conditions.

Although the identification of the desired environment and effective weather factor is vital in rainfed conditions, it should be emphasized that rainfed circumstances are varied over years and locations, so meticulous identification of well-adapted and stable genotypes is significant [31]. So, considering the detected significant GEI effect for DY and SY in the present study, the WAASB analysis was performed to identify stable genotypes based on DY × WAASB and SY × WAASB biplots. According to the literature review, there are narrow studies about yield stability analysis of grass pea and all of them utilized GGE biplot [21], non-parametric [20], and AMMI [9] in yield stability analysis. Meanwhile, it is possible to take advantage of WAASB as a new method derived from AMMI and BLUP methods for identifying superior grass pea genotypes for rainfed conditions. The WAASB method was successfully applied in several field crops [15, 18, 32, 33], but there have not been any reports about its usage in forage crops till now. However, our findings showed that Y × WAASB biplot could distinguish adaptable and stable genotypes in forage crops such as grass pea. Accordingly, for dry yield, the genotypes G5, G10, G11, G12, G13, and G15, and for seed yield, the genotypes G2, G3, G12, and G13 out of the studied grass pea genotypes were identified as stable ones. Since simultaneous evaluation of yield and yield stability is very important [34] in multi-environment analysis, ranking genotypes based on different weights of the yield and WAASB (WAASB/Y heatmap) can be more useful. Albeit, by assuming equal weights for both yield and stability index, the G13 had the best rank for dry yield and seed yield, but a plant breeder could designate differing weights for either yield or stability index through the key ability of WAASB analysis [12]. In this study, compared to yield performance, the stability of genotype was more desired, so weights of 40/60 (yield performance/stability) were chosen, and similarly, G13 was selected based on DY and SY.

Varietal recommendations would be more reliable if they were based on the mean performance and stability of multiple agronomically desirable traits [12], which are named MTSI. In MTSI, genotypes with the lowest MTSI are considered closer to the ideotype (ideal genotype) and thus selected. Here, G5 and G15 grass pea genotypes were selected, which also had average ranks (Fig. 5A and B) considering yield performance and WAASB index with 50/50 weights. Also, the selection differential was positive for all of the studied traits except for days to maturity, suggesting the effectiveness of the selection intensity [33]. About days to maturity in rainfed conditions, the development of early flowering plants with high yields could be calculated as a highlighted breeding objective to escape from water-deficient stress.

## Conclusions

Significant genotype × environment was found for majority of agro-morphological traits of grass pea specially for SY, and DY as economic parts of plant. This GEI highlighted importance of mutlti-environment trials in grass pea breeding programs. Partial least square regression method was found as reliable method for identifying effective monthly rainfall in ran-fed condition. Here in, this method emphasized on the critical role of rainfall during the initial growth stage (October and November) and highlighted the ongoing importance of monthly rainfall post-establishment, especially in May, for optimizing seed yield. In this study the WAASB stability parameter accompanied with either seed or dry yield (Y × WAASB) could effectively screened grass pea germplasm. Hence, regarding dry yield the genotypes G5, G10, G11, G12, G13, and G15 and regarding seed yield the genotypes G2, G3, G12, and G13 has been identified as superior and stable genotypes for examined rain-fed regions. A interesting findings of this research is screening grass pea germplasm by means of weighting each Y and WAASB items and so, a grass pea breeder could select interested genotype regarding breeding aim. For instance, with 50/50 of Y and WAASB the genotype G13 emerged as the top-ranked genotype for both dry and seed yield among the studied genotypes. In general, yield as dependent variable is correlated with several agro-morphological traits and therefore, plant breeders also try to incorporate several traits into a new genotype. Accordingly, it is recommended to evaluate performance of these traits along with yield. Here, by means of multiple trait selection index (MTSI) genotypes G5, G13, and G15 identified as promising superior genotypes which either have stable yield and also have acceptable performance regarding simoultenous considering agro-morphological traits.

## Supplementary Information

Supplementary Material 1.

Supplementary Material 2.

## Data Availability

The datasets generated during and/or analyzed during the current study are available through supplmentray file.
