# Supplementary material for: Deciphering genotype-by-environment interaction of grass pea genotypes under rain-fed conditions and emphasizing the role of monthly rainfall
Source: BMC Plant Biol. 2024 Jun 15;24:559. doi: 10.1186/s12870-024-05256-5 (PMC11613730; doi:10.1186/s12870-024-05256-5)
Supplement: Supplementary file 1 — Supplementary Material 1. [file 12870_2024_5256_MOESM1_ESM.docx]

Table S1. Physical and chemical properties of soil related with experimental sites

| Soil texture | Available K (ppm) | Available P (ppm) | N (%) | Organic carbon (%) | PH | Electric conductivity | Soil depth (cm) | Province | Location |
| --- | --- | --- | --- | --- | --- | --- | --- | --- | --- |
| Clay Loam‌ | 164 | 12 | 0.2 | 0.89 | 7.3 | 1.2 | 0-30 | Kohgiluyeh and Boyer-Ahmad | Gachsaran |
| Loam | 180 | 15 | 0.7 | 1.20 | 7.1 | 0.87 | 0-30 | Lorestan | Kuhdasht |
| Loam | 179 | 9 | 0.5 | 0.98 | 7.6 | 2.3 | 0-30 | Ilam | Mehran |
| Clay Loam | 145 | 13 | 0.5 | 0.88 | 7.4 | 1.9 | 0-30 | Ilam | Shirvan and Chardavol |

Table S2. Combined analysis of variance for studied agro-morphological traits of grass pea

| Source of variation | Degree of freedom | DF | DM | PH | PP | SP | WY | DY | SY |
| --- | --- | --- | --- | --- | --- | --- | --- | --- | --- |
| Environment | 11 | 42796.78^**^ | 39983.82^**^ | 19420.81^**^ | 9140.72^**^ | 74.71^**^ | 7398.94^**^ | 198.34^**^ | 30.33^**^ |
| Replication (Environment) | 24 | 30.62 | 143.66 | 90.15 | 95.88 | 1.05 | 43.31 | 3.45 | 0.45 |
| Genotype | 15 | 17.31^ns^ | 32.11 ^ns^ | 107.17 ^ns^ | 20.34 ^ns^ | 1.60 ^ns^ | 19.93 ^ns^ | 3.40^**^ | 0.73^*^ |
| Environment × Genotype | 165 | 15.03^**^ | 34.53^**^ | 73.30^**^ | 46.98^**^ | 1.21 | 16.00 | 1.18^**^ | 0.43^**^ |
| Error | 360 | 6.66 | 23.88 | 48.60 | 38.29 | 1.22 | 15.78 | 0.80 | 0.22 |

^*^ and ^**^ are significant in 1% and 5% probability level respectively. ^ns^ is non-significant. DF: days to flowering, DM: days to maturity, PH: plant height, PP: number of pods per plant, SP: number of seeds per pod, WY: wet fodder yield, DY: dry fodder yield, and GY: grain yield.
